# Epidemiology of Blood Stream Infection due to *Candida* Species in a Tertiary Care Hospital in Japan over 12 Years: Importance of Peripheral Line-Associated Candidemia

**DOI:** 10.1371/journal.pone.0165346

**Published:** 2016-10-31

**Authors:** Masahiro Ishikane, Kayoko Hayakawa, Satoshi Kutsuna, Nozomi Takeshita, Norio Ohmagari

**Affiliations:** 1 Department of Disease Control and Prevention Center, National Center for Global Health and Medicine, Tokyo, Japan; 2 Division of Global infectious diseases, Department of Infection and Epidemiology, Graduate School of Medicine, Tohoku University, Miyagi, Japan; Wadsworth Center, UNITED STATES

## Abstract

**Background:**

Candidemia is an important cause of mortality in healthcare settings. Peripheral lines are a source of candidemia, yet few studies have reported on the clinico-epidemiological features of candidemia due to peripheral-line associated blood stream infection (PLABSI).

**Methods:**

We conducted a single-centre retrospective cohort study of all patients with candidemia between 2002 and 2013. PLABSI was defined as the presence of at least one of the following: the presence of phlebitis or the resolution of clinical symptoms after peripheral-line withdrawal, with careful exclusion of an alternative explanation for bacteraemia. We described the epidemiology of candidemia and assessed predictive factors of PLABSI due to *Candida* spp., peripheral line-associated candidemia (PLAC), compared with non-PLAC.

**Results:**

A total of 301 episodes of candidemia, including 37 of PLAC, were diagnosed during the study period. Central-line associated blood stream infection, intra-abdominal infection, and infection of unknown source accounted for the remaining 233, 14, and 17 cases, respectively. The overall incidence rate of candidemia was 0.11/1000 patient-days. In multivariate analysis, cephalosporin exposure (odds ratio [OR] = 2.22, 95% CI 1.04–4.77), polymicrobial bacteraemia/fungaemia (OR = 2.87, 95% CI 1.02–8.10), and ID specialist consultation (OR = 2.40, 95% CI 1.13–5.13) were identified as independent predictors of PLAC. Although non-PLAC had a higher mortality, the length of hospital stay after candidemia was similar between the two groups and candidemia duration was longer in the PLAC group.

**Conclusion:**

PLACs are an important cause of candidemia in hospitalized patients. Appropriate identification and management of PLAC are crucial.

## Introduction

*Candida* is an important pathogen causing bloodstream infections in healthcare settings [1–5]. Candidemia is serious infection and its morbidity and mortality rates are high, with a reported overall mortality rate ranging from 25–60% [6–8]. In addition, candidemia is associated with prolonged hospitalization, resulting in substantially increased health care costs [6–8].

Central line-associated blood stream infection (CLABSI) is recognized as the main source of infection [9]; a central venous catheter (CVC) is present in at least 70% of non-neutropenic patients with candidemia at the time that the diagnostic blood culture is obtained [10–12]. CVCs pose a greater risk for vascular catheter-related bloodstream infections than short-term peripheral lines [13, 14]. In Japan, patients who require intravenous treatment are hospitalized. However, CVCs are not widely used and many patients receive parenteral solutions via peripheral lines [15–17]. Several previous studies show that peripheral line-associated blood stream infections (PLABSI) are caused not only by *Staphylococcus aureus* but also by *Candida* spp. [15–20]. The proportion of PLABSI secondary to *Candida* spp. is reported to range from 8.1% (5/62) to 14.7% (20/136) in Japan [15–19] and 0.2% to 1.1% in England [20]. Worldwide, there is a paucity of data on detailed clinical and epidemiological features of PLABSI, such as predictive factors. Moreover, studies on the epidemiology of candidemia in Asian countries, especially in Japan, are limited [21–23].

Therefore, we conducted this retrospective cohort study, which covered a 12-year period, to describe the epidemiology of candidemia in a tertiary care hospital in Japan, and to assess the epidemiology of PLABSI due to *Candida* spp.

## Materials and Methods

### Hospital setting and study design

We conducted a retrospective cohort study of all episodes of candidemia from January 2002 to December 2013 (12-year period). The setting was the National Center for Global Health and Medicine (NCGM), that has more than 800 inpatient beds and serves as a tertiary referral hospital for metropolitan Tokyo. This study was approved by the ethics committee of the NCGM (approval no: NCGM-G-001589-00) and was implemented in accordance with the provisions of the Declaration of Helsinki. Patient information was anonymized and deidentified prior to analysis, ant the need for patient consent was waived. This study was institutional review board-approved and patient consent was exempted because of retrospective nature.

### Data collection

All cases of candidemia were identified through the microbiological laboratory database. The parameters retrieved from patient records included the following: (i) demographics; (ii) immunosuppressive status (e.g. neutropenia at onset of candidemia, the use of immunosuppressive agents [including chemotherapy or steroid therapy], radiation therapy, transplantation; (iii) background and comorbid conditions (including Charlson’s scores [24]); (iv) recent healthcare-associated exposures (e.g. residence in a long term care facility [LTCF], previous hospitalization, invasive procedure and/or surgery in the 3 months preceding candidemia, the presence of a urinary catheter [≥ 2 days] and/or CVC [≥ 2 days] at the onset of candidemia, haemodialysis, intensive care unit stay during the current hospitalization episode prior to the onset of candidemia, and transfusion in the month preceding candidemia; (v) infection-related characteristics, including source of infection; (vi) recent exposure to antibiotic and antifungal therapy (for ≥ 3 days) within one month prior to the isolation of *Candida* spp.; (vii) the severity of illness, such as sepsis levels according to systemic inflammatory response syndrome criteria [25] and haematogenous dissemination; (viii) antifungal therapy against candidemia, including empirical or definitive antifungal therapy and source control; and (ix) outcome, including clinical failure, persistent candidemia for ≥ 3 days after initiation of antifungal therapy, in-hospital and 30-day/90-day mortality, discharged to a LTCF after being admitted from home, additional hospitalization within 6 months of completing candidemia therapy, and length of hospital stay after candidemia (excluding those who died), and duration candidemia. In addition, we reviewed referrals to an infectious disease (ID) specialist for management of candidemia or to an ophthalmology specialist for examination for endophthalmitis.

### Definitions of candidemia episode and other variables

An episode of candidemia was defined as isolation of *Candida* spp. from at least one peripherally taken blood culture in a patient with clinical signs and symptoms of infection [9]. Episodes were considered to be separate if they were caused by different species or occurred at least 30 days apart, with resolution of clinical features of infection and at least one negative blood culture in the intervening period [26]. Episodes detected within 48 hours of hospital admission were excluded as they were considered not to be hospital acquired, and it would be difficult to determine important parameters such as duration of candidemia.

CLABSI and intra-abdominal infection were defined according to the National Healthcare Safety Network Surveillance definition and the guidelines of the Infectious Diseases Society of America [27, 28]. Bloodstream infections related to peripherally inserted central catheters and port catheters were considered as CLABSI [9]. PLABSI was defined as the presence of at least one of the following conditions: (1) the presence of phlebitis, and/or (2) resolution of clinical symptoms after short-term peripheral line withdrawal with a careful exclusion of another focus of bacteraemia [15, 17, 18]. Phlebitis was diagnosed by the presence of at least two of the following signs on examination of the catheter insertion site: erythema, swelling, tenderness or pain, or warmth [18]. Peripheral line-associated candidemia (PLAC) was defined as PLABSI due to *Candida* spp. and non-PLAC as a source of infection other than other PLABSI (such as CLABSI, intra-abdominal infection, and unknown). Central line-associated candidemia was defined as CLABSI due to *Candida* spp. as described elsewhere [11].

Neutropenia was defined as an absolute neutrophil count < 0.5 × 10^9^ cells/L. Sepsis, severe sepsis, and septic shock were defined according to the Surviving Sepsis Campaign guidelines [25]. Empiric therapy was defined as administration of systemic antifungal drugs within 72 hours of the onset of candidemia, and definitive therapy was defined according to guideline of Infectious Diseases Society of America [9]. The time to antifungal therapy was determined as the time from when blood cultures which subsequently became positive for *Candida* spp. were obtained to the time of effective antifungal therapy initiation. Adequate source control was defined as removal of any pre-existing central or peripheral vein catheter or documented surgical or radiologic procedures to drain abscesses or other fluid collections (which were thought to be the source of candidemia) within 24 hours of the onset of candidemia. Time to central or peripheral vein catheter removal was determined based on medical record review. CVC removal was further classified into early removal (within 48 hours of candidemia) and replacement (removal with immediate re-insertion). Clinical failure was defined based on the presence of at least one of the followings: persistence of the clinical signs and symptoms of candidemia in the absence of another cause, and/or the same *Candida* spp. persistently detected on repeat blood cultures [29].

### Microbiological methods

*Candida* spp. were isolated from blood specimens using an automated broth microdilution system (MicroScan WlkAway; Siemens AG, Germany) [30] and identified using standard techniques. Antifungal susceptibility testing was performed using the commercially prepared colorimetric microdilution panel (ASTY; Kyokuto Pharmaceutical Industrial Co., Ltd.). During the study period, there were no changes to the microbiological identification and susceptibility testing process.

### Statistical analysis

Quantitative data were shown as the mean ± standard deviation (SD) or the median with interquartile range (Q1–Q3). Qualitative variables were expressed as absolute and relative frequencies. Categorical variables were compared using the χ^2^ test or Fisher’s exact test, whereas Student’s t-test or Mann-Whitney U test were applied for continuous variables. The number of episodes, distribution of the source of candidemia, and isolated *Candida* spp. were described.

Using logistic regression univariate analysis with odds ratios (OR) and 95% confidence intervals (CI), we compared demographic characteristics, clinical predictive factors, and outcomes between PLAC and non-PLAC cases. Potential predictive factors with a *P* value of < 0.10 in the univariate analysis or that were hypothesized a priori to be clinically or epidemiologically important were considered for inclusion in a multivariate model for predictive factors. Throughout the text, each of the percentages displayed represents the “valid percentage,” calculated with missing data excluded from the denominator. Statistical significance was defined as a 2-sided p-value of < 0.05. All statistical analyses were performed with SPSS Version 18 (SPSS Inc., Chicago, IL).

## Results

### Epidemiological description of candidemia between 2002 and 2013

**Fig 1:**
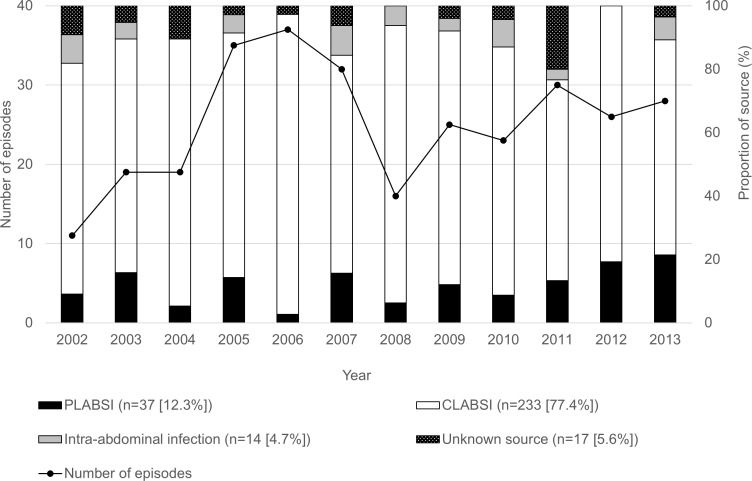
Number of episodes and distribution of source for candidemia, 2002–2013 (n = 301). The number of episodes of candidemia is indicated by solid lines. Bars express proportion of source for candidemia: black indicates PLABSI; white, CLABSI; grey, intra-abdominal infection; and dotted, unknown source.

A total of 301 episodes of candidemia from 293 patients were included. During the 12-year study period, the annual number of episodes ranged from 11 in 2002 to 37 in 2006. The overall incidence rate of episodes between 2005 and 2013 was 0.11/1000 patient-days and 1.74/1000 hospital admissions. The overall 30-day all-cause mortality was 26.9% (81/301). For source of infection, CLABSI, PLABSI, intra-abdominal infection, and unknown source consisted of 233 (77.4%), 37 (12.3%), 14 (4.7%), and 17 (5.6%), respectively. There was no patient identified who had both a CVC and peripheral episode of candidemia. The annual proportion of PLABSI ranged from 2.7% in 2006 to 21.4% in 2013. Although not statistically significant, the annual proportion of PLABSI tended to increase from 2010 to 2013 with a rate of increase of 68.5% (Fig 1).

**Fig 2:**
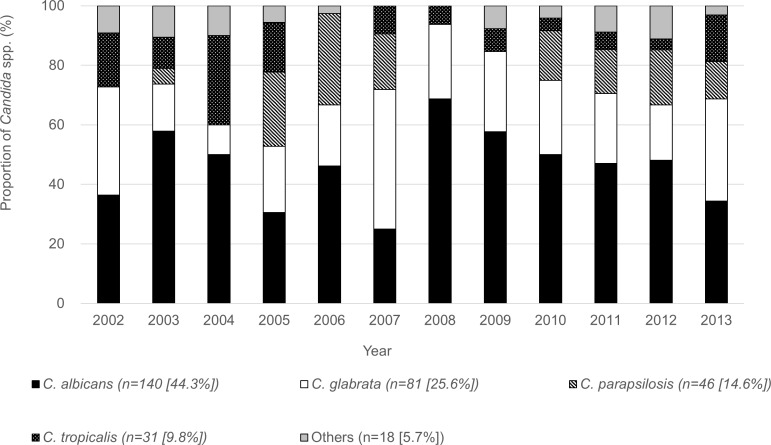
Distribution of isolated of Candida spp., 2002–2013 (n = 316). * * Includes 15 episodes of polymicrobial bacteraemia/fungaemia Bars express proportion of Candida spp.: black indicates C. albicans; white, C. glabrata; shaded, C. parapsilosis; dotted, C. tropicalis; and grey, others.

During this study period, a total of 316 *Candida* spp. were collected from the 301 episodes of candidemia, including 15 episodes of polymicrobial bacteraemia/fungaemia due to different species of *Candida* spp. and 14 due to pathogens other than *Candida* spp. There were 140 (44.3%) isolates of *C*. *albicans*, 81 (25.6%) of *C*. *glabrata*, 46 (14.6%) of *C*. *parapsilosis*, 31 (9.8%) of *C*. *tropicalis*, and 18 (5.7%) of other *Candida* spp., including *C*. *krusei* (n = 4), *C*. *guilliermondii* (n = 3), *C*. *lusitaniae* (n = 2), *C*. *dubliniensis* (n = 1), and unclassified (n = 8). The annual proportion of *C*. *albicans* reached its peak in 2008 (68.8%), and then showed a downward trend. The annual proportion of *C*. *parapsilosis* followed the same pattern (2006 peak, 30.8%), but tended to increase after 2010. While the annual proportion of *C*. *tropicalis* and *C*. *glabrata* peaked in 2004 (30.0%) and in 2007 (46.9%), respectively, then subsided, both showed a trend toward increasing after 2012 (Fig 2).

### Analysis of patients with PLAC and non-PLAC

The mean age of the study cohort was 69.9 ± 16.1 years and 204 (67.8%) were male. Although the patients in the two groups had a similar profile of chronic conditions, patients in the PLAC group tended to have more solid-organ cancers and chronic heart disease. The non-PLAC group had significantly more healthcare-associated exposures, such as urinary catheter insertion, CVC insertion, and transfusion. While there was no significant difference in previous exposure to antibiotic drugs overall or to antifungal drugs in particular, cephalosporin exposure was higher in the group with PLAC. No difference of isolated *Candida* spp. was observed in two groups, but the PLAC group had significantly more episodes of polymicrobial bacteraemia/fungaemia than the non-PLAC group. Non-PLAC episodes had a tendency to increase with severe sepsis, and were associated with acute renal failure.

Regarding treatment, no difference was observed in empirical/definitive regimens and treatment duration between the two groups. ID consultations were more frequent in the PLAC than in the non-PLAC group. The in-hospital and 30-day/90-day mortality of non-PLAC was higher than that of PLAC (*P* = 0.017). The length of hospital stay after candidemia was similar between the two groups (41 days in PLAC vs 36 days in non-PLAC), while the duration of candidemia was longer in PLAC (9 days in PLAC vs 6 days in non-PLAC). Patients with PLAC tended to be discharged to an LTCF after being admitted from home (Table 1).

**Table 1:** Univariate analysis of candidemia: PLAC versus non-PLAC, 2002–2013 (n = 301).

Category	Variable	PLAC (n = 37)		Non-PLAC^a^ (n = 264)		OR (95% CI)		*P* value
Demographics	Mean age (years) ± SD	68.4	± 21.0	69.9	± 15.8			0.68
	Females	11	(29.7)	86	(32.6)	0.88	(0.41–1.86)	0.73
Immunocompromised status	HIV infection	3	(8.1)	7	(2.7)	3.24	(0.809–13.12)	0.11
	Neutropenia (<0.5 × 10^9^ cells/L) at onset	0	(0.0)	7	(2.7)			0.60
	Chemotherapy in the past month	7	(18.9)	45	(17.0)	1.14	(0.47–2.75)	0.78
	Steroid therapy in the past month	7	(18.9)	48	(18.2)	1.05	(0.44–2.53)	0.91
	Radiation therapy in the past month	3	(8.1)	17	(6.4)	1.28	(0.36–4.61)	0.72
	Transplantation in the past month	1	(2.7)	3	(1.1)	2.42	(0.25–23.86)	0.41
Background and comorbid conditions on admission	Dependent functional status	18	(48.6)	115	(43.6)	1.23	(0.62–2.44)	0.56
	Age adjusted Charlson's weighted index co-morbidity score (6), mean ± SD	6.4	± 2.3	6.9	± 3.2			0.21
	Diabetes mellitus	10	(27.0)	72	(27.3)	0.99	(0.46–2.14)	0.98
	Solid-organ cancer within last 1 year	9	(24.3)	107	(40.5)	0.47	(0.21–1.04)	0.058
	Haematological malignancy within last 1 year	3	(8.1)	24	(9.1)	0.88	(0.25–3.09)	1.00
	Chronic kidney disease stage V	1	(2.7)	17	(6.4)	0.40	(0.05–3.13)	0.71
	Liver diseases	0	(0.0)	15	(5.7)			0.23
	Chronic heart disease	12	(32.4)	50	(18.9)	2.05	(0.97–4.37)	0.057
	Chronic obstructive pulmonary disease	3	(8.1)	33	(12.5)	0.62	(0.18–2.13)	0.59
	Cerebrovascular disease	11	(29.7)	57	(21.6)	1.54	(0.72–3.30)	0.27
	Dementia	4	(10.8)	19	(7.2)	1.56	(0.50–4.88)	0.50
	Connective tissue disease	3	(8.1)	15	(5.7)	1.47	(0.40–5.32)	0.47
	Peptic ulcer disease	2	(5.4)	24	(9.1)	0.57	(0.13–2.52)	0.75
	Peripheral vascular disease	0	(0.0)	4	(1.5)			1.00
	Hemiplegia	5	(13.5)	22	(8.3)	1.72	(0.61–4.86)	0.30
Recent health care-associated exposures before/onset of candidemia	Resided at an LTCF in the past 3 months	1	(2.7)	14	(5.3)	0.50	(0.06–3.89)	0.70
	Hospitalized in the past 3 months	17	(45.9)	122	(46.2)	0.99	(0.50–1.97)	0.98
	Invasive procedure/surgery in the past 3 months	13	(35.1)	92	(34.8)	1.01	(0.49–2.08)	0.97
	Tracheotomy in the past 3 months	4	(10.8)	29	(11.0)	0.98	(0.33–2.97)	1.00
	Urinary catheters (for ≥ 2 days) at onset of candidemia	18	(48.6)	174	(65.9)	0.49	(0.25–0.98)	0.041
	CVC (for ≥ 2 days) at same onset	8	(21.6)	237	(89.8)	0.03	(0.01–0.08)	<0.001
	Median days of CVC prior to onset of candidemia (IQR)	0	(0–0)	13	(7–24)			<0.001
	Undergoing haemodialysis in the past month	1	(2.7)	21	(8.0)	0.32	(0.04–2.46)	0.50
	Transfusion in the past month	12	(32.4)	150	(57.0)	0.36	(0.17–0.75)	0.005
	ICU stay in current hospitalization before onset of candidemia	3	(8.1)	35	(13.3)	0.58	(0.17–1.98)	0.60
	Median hospital days prior to the onset of candidemia (IQR)	33	(13–75)	35	(20–65)			0.68
Exposure to antibiotic therapy (for ≥3 days) prior to isolation of *Candida* spp.	Overall	36	(97.3)	249	(94.3)	2.17	(0.28–16.92)	0.45
	Penicillins^b^	16	(43.2)	113	(42.8)	1.02	(0.51–2.04)	0.96
	Cephalosporins^c^	22	(59.5)	116	(43.9)	1.87	(0.93–3.77)	0.076
	Carbapenems	18	(48.6)	133	(50.4)	0.93	(0.47–1.86)	0.84
	Fluoroquinolones	8	(21.6)	63	(23.9)	0.88	(0.38–2.02)	0.76
	Aminoglycosides	6	(16.2)	26	(9.8)	1.77	(0.68–4.64)	0.24
	Trimethoprim-sulfamethoxazole	3	(8.1)	30	(11.4)	0.69	(0.20–2.38)	0.78
	Clindamycin	4	(10.8)	30	(11.4)	0.95	(0.31–2.86)	1.00
	Metronidazole	1	(2.7)	24	(9.1)	0.28	(0.04–2.12)	0.34
	Glycopeptides	12	(32.4)	80	(30.3)	1.10	(0.53–2.31)	0.79
Exposure to antifungal therapy (for ≥3 days) prior to isolation of *Candida* spp.	Over all	3	(8.1)	35	(13.3)	0.58	(0.17–1.98)	0.60
	Fluconazole	1	(2.7)	11	(4.2)	0.64	(0.08–5.10)	1.00
	Micafungin	2	(5.4)	20	(7.6)	0.70	(0.16–3.11)	1.00
	Voriconazole	1	(2.7)	1	(0.4)	7.31	(0.45–119.37)	0.23
	Liposomal amphotericin b	1	(2.7)	3	(1.1)	2.42	(0.25–23.86)	0.41
	Itraconazole	1	(2.7)	7	(2.7)	1.02	(0.12–8.53)	1.00
Microbiology	*Candida* species:							
	*C*. *albicans*	16	(43.2)	124	(47.0)	0.86	(0.43–1.72)	0.67
	*C*. *glabrata*	11	(29.7)	70	(26.5)	1.17	(0.55–2.50)	0.68
	*C*. *parapsilosis*	5	(13.5)	41	(15.5)	0.85	(0.31–2.31)	0.75
	*C*. *tropicalis*	3	(8.1)	28	(10.6)	0.74	(0.21–2.58)	0.78
	Others^d^	4	(10.8)	14	(5.3)	2.17	(0.67–6.97)	0.25
	Polymicrobial bacteraemia/fungaemia^e^	7	(18.9)	22	(8.3)	2.57	(1.01–6.51)	0.041
	Previous *Candida* colonization within a week before candidemia	13	(35.1)	88	(33.3)	1.08	(0.53–2.23)	0.83
Severity of illness indices at the time of candidemia	Sepsis	17	(45.9)	78	(29.5)	2.03	(1.01–4.08)	0.044
	Severe sepsis	9	(24.3)	109	(41.3)	0.46	(0.21–1.01)	0.048
	Septic shock	4	(10.8)	40	(15.2)	0.68	(0.23–2.01)	0.62
	Reduced consciousness	2	(5.4)	37	(14.0)	0.35	(0.08–1.52)	0.19
	Acute mechanical intubation/ventilation	2	(5.4)	31	(11.7)	0.43	(0.10–1.87)	0.40
	Developed acute renal failure	3	(8.1)	62	(23.5)	0.29	(0.09–0.97)	0.033
	Developed acute liver injury	8	(21.6)	93	(35.2)	0.51	(0.22–1.15)	0.10
	Chorioretinitis	4	(10.8)	29	(11.0)	0.98	(0.33–2.97)	1.00
Therapy	Empirical antifungal therapy within the 72 hours of the onset of candidemia							
	Fluconazole	7	(18.9)	79	(29.9)	0.55	(0.23–1.30)	0.17
	Micafungin	25	(67.6)	146	(55.3)	1.69	(0.81–3.49)	0.16
	Voriconazole	0	(0.0)	1	(0.4)			1.00
	Liposomal amphotericin b	0	(0.0)	11	(4.2)			0.37
	None	5	(13.5)	26	(9.8)	1.43	(0.51–3.99)	0.49
	Definitive antifungal therapy							
	Fluconazole	17	(45.9)	116	(43.9)	1.08	(0.54–2.16)	0.82
	Micafungin	14	(37.8)	106	(40.2)	0.90	(0.45–1.84)	0.79
	Voriconazole	0	(0.0)	3	(1.1)			1.00
	Liposomal amphotericin b	1	(2.7)	12	(4.5)	0.58	(0.07–4.62)	1.00
	None	5	(13.5)	26	(9.9)	1.43	(0.51–3.99)	0.49
	Change the antifungal drugs due to clinical failure	6	(16.2)	26	(9.8)	1.77	(0.68–4.64)	0.24
	Median treatment duration days (IQR)	17	(12.3–29.8)	16	(9–24)			0.33
	Adequate source control	25	(67.6)	222	(84.1)	0.39	(0.18–0.85)	0.014
	CVC removal	0	(0.0)	216	(81.8)			<0.001
	Early CVC removal (≤48 hours)	0	(0.0)	176	(66.7)			<0.001
	CVC replacement	0	(0.0)	59	(22.3)			<0.001
	Peripheral-line removal	25	(67.6)	0	(0.0)			<0.001
	Intra-abdominal drainage	0	(0.0)	6	(2.3)			1.00
	Consultation to ID specialist	23	(62.2)	104	(39.4)	2.53	(1.24–5.14)	0.009
	Consultation to Ophthalmologist	21	(56.8)	133	(50.4)	1.29	(0.65–2.59)	0.47
Outcome	Clinical failure	6	(16.7)	77	(29.2)	0.47	(0.19–1.17)	0.099
	Persistent candidemia for ≥72 hours of therapy	33	(89.2)	232	(87.9)	1.14	(0.38–3.42)	0.82
	In-hospital mortality	10	(27.0)	118	(44.7)	0.46	(0.21–0.99)	0.042
	30-day mortality	4	(10.8)	78	(29.5)	0.29	(0.10–0.84)	0.017
	Early (<72 hours)	1	(2.7)	14	(5.3)	0.50	(0.06–3.89)	0.70
	Non-early (days 3–30)	3	(8.1)	64	(24.2)	0.28	(0.08–0.93)	0.033
	90-day mortality	8	(21.6)	113	(42.8)	0.37	(0.16–0.84)	0.014
	Discharged to LTCF after being admitted from home	16	(43.2)	71	(26.9)	2.07	(1.02–4.19)	0.040
	Additional hospitalizations in 6 months after completed candidemia therapy	5	(13.5)	45	(17.0)	0.76	(0.28–2.06)	0.59
	Median total LOS days (IQR)	91	(51.8–137.5)	79	(50–202.9)			0.52
	Median LOS after candidemia day (IQR)	40.5	(24–126.5)	36	(15.3–66.5)			0.31
	Median LOS after candidemia excluding those who died days (IQR)	24.5	(0–58.8)	18	(0–53.8)			0.21
	Median ICU LOS after candidemia days (IQR)	0	(0–0)	0	(0–0)			0.40
	Median duration candidemia days (IQR)	8.5	(5–12)	6	(1–11)			0.064

Unless otherwise stated, data are presented as n (%). PLAC, peripheral-line associated blood stream infection due to *Candida* species; OR, odds ratio; CI, confidence interval; CLABSI, central line-associated blood stream infection; SD, standard deviation; LTCF, long term care facility; IQR, interquartile range; CVC, central venous catheter; ID, infectious disease; LOS, length of hospital stay.

^a^CLABSI (n = 233), Intra-abdominal infection (n = 14), Unknown source (n = 17).

^b^Included ampicilline, sulbactam/ampicilline, piperacillin, and tazobactam/piperacillin.

^c^Included ceftriaxone, ceftazidime, and cefepime.

^d^Other *Candida* species included *C*. *guilliermondii* (2 in PLAC, 1 in Non-PLAC), *C*. *lusitaniae* (2 in Non-PLAC), *C*. *krusei* (4 in Non-PLAC), *C*. *dubliniensis* (1 in Non-PLAC) and unclassified (2 in PLAC, 6 in Non-PLAC).

^e^Polymicrobial bacteraemia/fungaemia were included due to different species of *Candida* spp. (2 in PLAC, 13 in Non-PLAC) and due to pathogens other than *Candida* spp. (5 in PLAC, 9 in Non-PLAC).

### Predictive factors of PLAC and non-PLAC

In the univariate analysis, PLAC was significantly negatively associated with the presence of urinary catheters or transfusion, and was significantly associated with polymicrobial bacteraemia/fungaemia and ID consultations required. Independent predictive factors for PLAC identified on multivariate analysis were previous cephalosporins exposure (*P* = 0.040; OR = 2.22; 95% CI = 1.04–4.77), polymicrobial bacteraemia/fungaemia *(P* = 0.046; OR = 2.87; 95% CI = 1.02–8.10), and ID consultations obtained (*P* = 0.023; OR = 2.40; 95% CI = 1.13–5.13). In contrast, urinary catheters (*P* = 0.040; OR = 0.45; 95% CI = 0.21–0.96) and transfusions (*P* = 0.039; OR = 0.44; 95% CI = 0.20–0.96) were significantly negatively associated with PLAC (Table 2).

**Table 2:** Multivariate analysis for predictive factors of PLAC compared to non-PLAC, 2002–2013 (n = 301).

	No. (%) of patients with:				Univariate analysis			Multivariate analysis			
	PLAC		Non-PLAC								
Variable	(n = 37)		(n = 264)		Crude OR (95% CI)		*P* value	Adjusted OR (95% CI)			*P* value
Solid-organ cancer within the last year	9	(24.3)	107	(40.5)	0.47	(0.21–1.04)	0.058	0.48		(0.21–1.13)	0.092
Chronic heart disease	12	(32.4)	50	(18.9)	2.05	(0.97–4.37)	0.057	2.07		(0.90–4.74)	0.087
Urinary catheter (for ≥2 days) at onset of candidemia	18	(48.6)	174	(65.9)	0.49	(0.25–0.98)	0.041	0.45		(0.21–0.96)	0.040
Transfusion in the month preceding candidemia	12	(32.4)	150	(57.0)	0.36	(0.17–0.75)	0.005	0.44		(0.20–0.96)	0.039
Polymicrobial bacteraemia/fungaemia^a^	7	(18.9)	22	(8.3)	2.57	(1.01–6.51)	0.041	2.87		(1.02–8.10)	0.046
Cephalosporin^b^ exposure (for ≥3 days) in the month preceding candidemia	22	(59.5)	116	(43.9)	1.87	(0.93–3.77)	0.076	2.22		(1.04–4.77)	0.040
Consultation to an ID specialist	23	(62.2)	104	(39.4)	2.53	(1.24–5.14)	0.009	2.40		(1.13–5.13)	0.023

Unless otherwise stated, data are presented as n (%). PLAC, peripheral-line associated blood stream infection due to *Candida* spp.; OR, odds ratio; CI, confidence interval; ID, infectious disease.

^a^Polymicrobial bacteraemia/fungaemia were included due to different species of *Candida* spp. (2 in PLAC, 13 in Non-PLAC) and due to pathogens other than *Candida* spp. (5 in PLAC, 9 in Non-PLAC).

^b^Includes ceftriaxone, ceftazidime, cefepime.

## Discussion

Our study showed that the overall incidence rate of candidemia was 0.11/1000 patient-days and 1.74/1000 hospital admissions. Our incidence rate was higher than those reported in recent studies conducted in Italy (1.19–1.50/1000 hospital admissions) [2, 31], Turkey (0.95/1000 hospital admissions) [32], Spain (0.92/1000 hospital admissions) [33], Finland (0.026–0.03/1000 hospital admissions) [34], the USA (0.16–0.33/1000 hospital admissions), and Australia (0.23/1000 hospital admissions) but lower than that reported in Brazil (0.54/1000 patient-days) [35–37]. Unlike in Western European countries, where there is an observed trend toward increased incidence rates of candidemia, our incidence rate did not significantly increase over the study period.

Although *Candida* spp. was not reported as a causative organism of PLABSI in Spain [18], a study from England showed that the proportion of *Candida* spp. among PLABSI was 0.2% in non-teaching hospitals and 1.1% in teaching hospitals [20]. However, some studies in Japan showed more frequent isolation of *Candida* spp. among PLABSI (8.1% to 14.7%) [15–19]. This result might reflect the difference in practice of using peripheral lines, not CVCs, to administer parenteral solutions in Japan. Consistent with other previous studies [38, 39], the annual proportion of *C*. *albicans* showed a decreasing trend in our study, and *C*. *albicans* was isolated as frequently as *C*. *glabrata* in 2013 (i.e. 34.4%). As a previous study pointed out, different frequencies of isolation of each *Candida* spp. depend on local factors, such as the population involved, geographical region, and previous anti-fungal exposure [40]. As our hospital is a tertiary referral center, patients’ population comprised mixed patients such as patients admitted through emergency room, patients with sold and hematological malignancies, and patients undergoing surgeries. Our trend is similar to trend of the previous nation-wide survey in Japan conducted in 14 various hospitals [41]. Therefore, our results might reflect the trend of mixed patients’ population in Japan.

While no difference of *Candida* spp. was observed between the two groups, PLAC had more episodes of polymicrobial bacteraemia/fungaemia than non-PLAC. This may be due to heavier contamination of peripheral lines than central lines. Peripheral lines were frequently inserted in the emergency department, or in hospital wards where nurses and trainees occasionally inserted peripheral lines without adequate infection control procedures. These issues might have contributed to PLAC. The United States Centers for Disease Control and prevention recommend that peripheral lines inserted in emergency situations should be removed or changed in hospital wards within the first 48 hours of admission and every 72–96 hours thereafter, irrespective of the presence of infection [42]. In our hospital, only 70% ethanol (not chlorhexidine gluconate) was used as antiseptic skin preparation, and the exchange of peripheral lines was performed at least every 96 hours or earlier if phlebitis developed. This practice did not change over this study period.

PLAC episodes were more strongly associated with cephalosporin exposure than non-PLAC. These results might reflect the difference of severity of illness among the two groups. Although we used severity of sepsis as an indicator of severity [25], non-PLAC had a tendency to increase with severe sepsis and acute renal failure. Further, a greater proportion of patients who developed non-PLAC received broad-spectrum antibiotic therapy, such as a carbapenem (133/254: 50.4% in non-PLAC vs 18/37: 48.6% in PLAC). In fact, the presence of urinary catheters and having received a transfusion were associated with non-PLAC; these results indicate that non-PLAC was more serious than PLAC. Remarkably, ID consultation was requested more frequently in PLAC than non-PLAC. This might reflect the difficulty of diagnosing PLAC, and need for the consultation of ID specialists. The increasing number of PLAC episodes since 2010 might reflect the strengthened ID consulting system since 2011 (e.g. increasing the number of ID specialists).

Although in-hospital and 30-day/90-day mortality of non-PLAC was higher than that of PLAC, length of hospital stay after candidemia was similar between the two groups, and duration of candidemia in PLAC was longer. These results indicate that PLAC was clinically important, and early detection and treatment of PLAC could save health care costs in hospital settings.

As a limitation, the present study was conducted only at a single centre. Therefore, our results might be influenced by the local clinical management practices and infection control policies. However, the candidemia incidence rate reported in the present study is similar to that reported in a multicentre analysis in Japan [43]. In addition, due to the retrospective nature of the study, we were unable to collect information such as duration of peripheral line insertion.

In conclusion, our study is the first epidemiological study related PLAC to reveal that peripheral lines are an important source of candidemia. We found that PLAC was associated with ID consultation requests, polymicrobial bacteraemia/fungaemia, and exposure to cephalosporins. Although the recent rise in PLAC may be due to enhanced detection by ID specialists, this study has led to a better understanding of candidemia and highlights the potential problem in hospital setting: that not only central lines but also peripheral lines may be source of candidemia. Also, the high hospital mortality (27.0%), long length of hospital stay after candidemia, and long duration of candidemia associated with PLAC are clinically important. Further studies are warranted to investigate the current situation and impact of candidemia worldwide, as appropriate identification of PLAC should lead to effective and efficient control systems to prevent the spread of candidemia.
